# Household cereal crop harvest and children’s nutritional status in rural Burkina Faso

**DOI:** 10.1186/s12940-017-0258-9

**Published:** 2017-06-20

**Authors:** Kristine Belesova, Antonio Gasparrini, Ali Sié, Rainer Sauerborn, Paul Wilkinson

**Affiliations:** 1 Department of Social and Environmental Health Research, London School of Hygiene & Tropical Medicine, 15-17 Tavistock Place, London, WC1H 9SH UK; 2 Centre de Recherche en Santé de Nouna, Rue Namory Keïta, Nouna, Kossi province, Boucle du Mouhoun region Burkina Faso; 3 Institute of Public Health, Heidelberg University, Im Neuenheimer Feld, Heidelberg, 324 69120 Germany

**Keywords:** Climate change, Malnutrition, Undernutrition, MUAC, Agriculture, Crops, Food security, Children’s health, Environmental epidemiology

## Abstract

**Background:**

Reduction of child undernutrition is one of the Sustainable Development Goals for 2030. Achievement of this goal may be made more difficult in some settings by climate change through adverse impact on agricultural productivity. However, there is only limited quantitative evidence on the link between household crop harvests and child nutrition. We examined this link in a largely subsistence farming population in rural Burkina Faso.

**Methods:**

Data on the middle-upper arm circumference (MUAC) of 975 children ≤5 years of age, household crop yields, and other parameters were obtained from the Nouna Health and Demographic Surveillance System. Multilevel modelling was used to assess the relationship between MUAC and the household crop harvest in the year 2009 estimated in terms of kilocalories per adult equivalent per day (kcal/ae/d).

**Results:**

Fourteen percent of children had a MUAC <125 mm (a value indicative of acute undernutrition). The relationship between MUAC and annual household food energy production adjusted for age, sex, month of MUAC measurement, household wealth, whether a household member had a non-agricultural occupation, garden produce, village infrastructure and market presence, suggested a decline in MUAC below around 3000 kcal/ae/d. The mean MUAC was 2.49 (95% CI 0.45, 4.52) mm less at 1000 than at 3000 kcal/ae/d.

**Conclusions:**

Low per capita household crop production is associated with poorer nutritional status of children in a rural farming population in Burkina Faso. This and similar populations may thus be vulnerable to the adverse effects of weather on agricultural harvest, especially in the context of climate change.

**Electronic supplementary material:**

The online version of this article (doi:10.1186/s12940-017-0258-9) contains supplementary material, which is available to authorized users.

## Background

Reducing child undernutrition and hunger is at the top of the global development agenda. It is the primary objective of the Sustainable Development Goal (SDG) No. 2 (Target 2: “by 2030 end all forms of malnutrition, including achieving by 2025 the internationally agreed targets on stunting and wasting in children under five years of age [..]” [1]) and is reflected in the policy agendas of many development agencies [2]. Malnutrition is estimated to be responsible for over a fifth of the global disease burden in children under five years of age [3, 4] and for 45% of the 5.9 million deaths in children under five in 2015 [5]. Legacy effects of childhood undernutrition may also continue into adulthood. Adults undernourished in childhood are more susceptible to infectious [6] and chronic disease [7], have lower economic productivity [8], and are more likely to have compromised cognitive development [9].

While the proportion of undernourished children in developing regions dropped from 23.3% in 1990–1992 to 12.9% in 2014–2016, the rate of improvement over time has been slowing [10]. Climate change impacts on agricultural productivity may further challenge the achievement of SDG 2. Some analyses suggest that, in some settings, it could even lead to the reversal of the recent trend of decreasing undernutrition [11–16].

Household food security, a key determinant of children’s nutritional status [4], is widely recognised to have four key dimensions: food availability (sufficient quantity of food of adequate quality), food access (adequate resources to acquire appropriate foods), utilization (sufficient nutrient and energy intake, resulting from appropriate food preparation, diet, intra-household food distribution, feeding practices, good care), and stability (access to adequate food at all times) [17, 18]. In subsistence farming populations the agricultural harvest is both a source of food and of income for food purchases [19], yet its yield may vary appreciably because of variations in weather and other factors. Such populations therefore have potential vulnerability in relation to at least three of the four pillars of food security: food availability, access, and stability.

What is unclear, however, is the degree to which reduced household crop yields result in compromised nutrition. Studies in different settings provide differing results on the association of children’s nutritional status with household food crop production [20–24], possibly reflecting effect of context-specific factors and variation in vulnerability across study populations (e.g., previously suggested to differ by the level of income [25–27], diversity of the cultivated crops [28, 29], gender [30], and age [24, 31]).

In this paper we report a study examining the relationship between children’s nutritional status, measured by middle-upper arm circumference (MUAC), and household cereal crop production in a largely subsistence farming population of rural Burkina Faso.

## Methods

### Study area and population

Burkina Faso is a land-locked low-income country in West Africa, which in 2009 was ranked 6^th^ from bottom in terms of the Human Development Index [32]. In 2009 46.7% of the population lived below the poverty line of US$1.25 per person per day. 73.5% of the population is rural and relies on rain-fed agriculture [33].

**Fig. 1:**
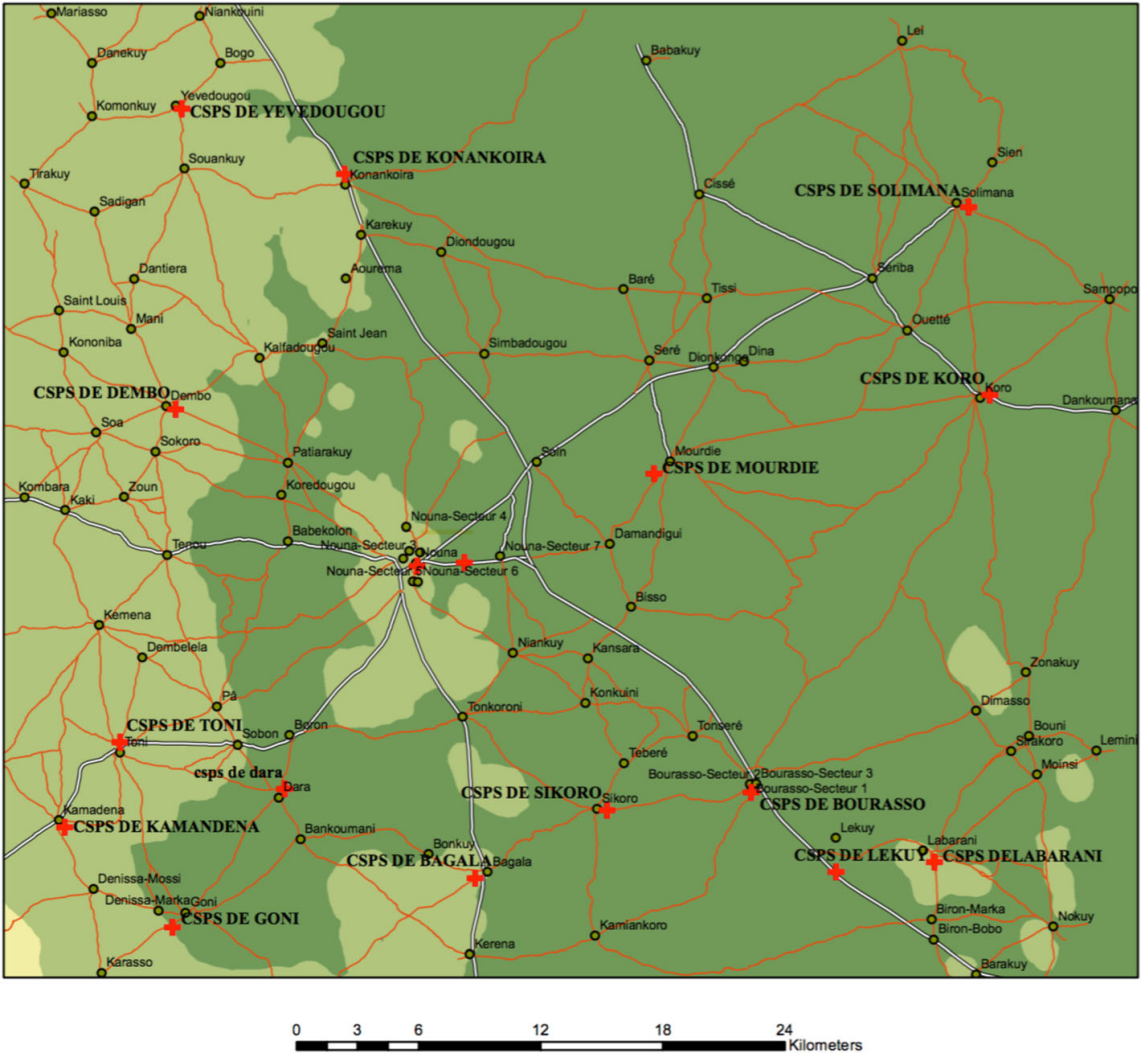
Map of Nouna HDSS villages

The study was conducted within the population of the Nouna Health and Demographic Surveillance System (HDSS) in the Kossi province of Western Burkina Faso (Fig. 1), which has been surveyed by the Centre de Recherche en Santé de Nouna (CRSN) since 1992. The Kossi province is classified as a dry orchard savannah, and receives on average 685 mm of rainfall per year [34, 35]. The single agricultural production season lasts during the rainy season starting in June and ending in October [35]. Agricultural productivity in the Kossi province in the year of study (2009/10) was close to the average for the past 30 years. Cereals constitute 72.2% of the average daily kilocalorie (kcal) consumption in the country as a whole [36], while other food groups contribute much smaller proportions: vegetables 0.44%, meat 3.18%, and fish 3.45% [36].

The Nouna HDSS population almost exclusively relies on rain-fed crop produce [34, 35, 37]. Ninety-eight per cent of the population cultivate crops for food, and in most cases at levels no greater than required to meet household needs. The main food crops cultivated are millet, sorghum, fonio, maize, and rice. Farmers also grow cotton, sesame, and peanuts. Although here these three are referred to as cash crops, they are mostly gown to meet day-to-day household expenses (e.g., health care and schooling fees) from small scale sales. Sesame and peanuts can also serve as food for the household.

Here we follow the Nouna HDSS definition of a household as a socio-economic unit whose members are usually, but not necessarily, related by family ties; household members live together, share resources, and jointly meet their food and other vital needs under the authority of a single person, referred to as the head of the household [38]. The head of the household oversees agricultural activities undertaken by household members in the crop fields. Crop cultivation and harvest are mostly performed by men. In some households women may participate in selected stages of agricultural work, such as sowing and weeding. Women frequently maintain gardens where they cultivate vegetables, fruit, and herbs. Women are also responsible for grain processing, food preparation, and sales of their garden produce and food products.

Previous studies indicate high levels of child undernutrition in the Nouna HDSS population. In a sample of 460 children 6–31 months of age taken in June 2009 35% were underweight (weight-for-age z-score < −2 standard deviations (SD)), 30% stunted (length-for-age z-score < −2 SD), and 26% had wasting (weight-for-length z-score < −2 SD) [39]. The mean MUAC in this sample was 140 mm with the standard deviation of 12 mm [39].

### Study sample

The study was of children aged 0*–*5 years who had undergone routine measurement of MUAC as part of the HDSS survey protocol in the year 2010. Such measurements were made in 604 households (5.83% of all 10,364 households in the Nouna HDSS population). We selected for our analysis the 545 households (containing 975 children ≤5 years in 52 villages in the Nouna HDSS area and Nouna town) that were involved in food and/or cash crop production. Although these households were not selected by random sampling, inclusion of households in the MUAC measurement survey was non-selective with regard to household characteristics, and the children ≤5 years in our sample have similar demographic and socio-economic characteristics to those of children ≤5 years in the wider population, except with regard to age (Additional file 1: Table S1). The MUAC measurement protocol specifically targeted infants <6 months, who are therefore substantially over-represented in the sample.

### Data

The data assembled for our analyses were as follows:

#### Middle upper arm circumference (MUAC) – the main outcome variable

We used the MUAC measurements collected by the CRSN survey team between March and August 2010. MUAC values greater than 5 standard deviations of the mean (i.e., outside the range 67 to 218 mm) were considered implausible and excluded from analysis [40–42]. MUAC is a commonly used anthropometric measure indicative of children’s short-term nutritional status and shown to be highly correlated with weight change [43]. In the age group of 6 months–5 years MUAC values <125 mm are commonly interpreted as indicative of moderate acute undernutrition and values <115 mm of severe acute undernutrition [44–47].

#### Food energy value of the household cereal crop harvest in 2009 – the explanatory factor of primary interest

Data on the annual quantity of each food and cash crop harvested by a household in 2009 (i.e., in the year before MUAC measurements) were recorded in the HDSS socio-economic census survey. Quantities described in such terms as *tin*, *can* or *charette* were converted into kilograms using conversion factors provided by the CRSN. From these we computed two measures:

1. The energy value of average daily household *food cereal* crop produce in kilocalories/adult equivalent/day (kcal/ae/d), E_f_ = $\sum_i\left({h}_i\times {c}_i\right)$/ae/356.25
2. The energy value of average daily household *food and cash* crops combined (kcal/ae/d), E_fc_ = $\Big(\sum_i\left({h}_i\times {c}_i\right)$ + ${c}_{millet}\times \sum_j\left({h}_j\times {p}_j\right)/{p}_{millet}\Big)$ /ae/356.25

where

- i – food crop: millet, maize, sorghum, fonio, rice
- j – cash crop: cotton, peanuts, sesame
- h – weight (kg) of the crop
- c – caloric value of 1 kg of the food crop i [48]
- p – market price of 1 kg of peanuts, sesame, or millet in December 2009 in Nouna market prices or 1 kg of cotton in SOFITEX [49, 50]
- ae – number of adult equivalents (ae) in the household, using weights to reflect differences in physiological food energy needs by age and sex (a 30–60 years old male was given the weight of 1) [51, 52].

Thus, our main measure of food availability, E_f_, was based on the cereal crop harvest and does not consider the usually modest but unquantified food energy from *garden* produce (vegetables, fruit, herbs etc.). (Possible differences related to the availability of garden produce were captured by adjusting for the presence of the garden produce as a binary measure in analysis – see below).

The E_fc_ indicates the maximum potential food energy that households could acquire using all income from cash crops to purchase millet (if they chose to do so) and consuming all of their food crop harvest.

The value of ae was calculated following the method suggested by Smith and Subandoro [50], where age and sex-specific energy requirements of all household members are standardised against the energy requirements of a 30–60 years old male, but using the latest guidelines on energy and protein requirements [51, 52], and assuming moderate activity levels for household members.

The kcal value of cash crop produce was estimated as the amount that would be available if the household sold all their cash crop harvest (cotton to SOFITEX in 2009, peanuts and sesame on the Nouna market in December 2009) and used the entire income from these sales to purchase millet on the Nouna market in December 2009. Data on the price of millet, sesame, and peanuts were collected by CRSN from the Nouna town market in December 2009, and that of cotton from the cotton producer’s price reports for 2009/10 provided by the Societé Burkinabé des Fibres et Textiles (SOFITEX), the biggest cotton company in Burkina Faso controlling cotton production in the Kossi province [49, 50].

#### Individual, household, and village-level co-variates

Data on child’s age, sex, and month of MUAC measurement, as well as household characteristics (number of people in the household, age categories of household members, household members’ occupations, housing conditions, and assets) were obtained from the HDSS surveys.

As a measure of socio-economic status we used the household wealth index of Schoeps et al. [53], re-coded into quartiles. The wealth index reflects household asset ownership (e.g., means of transport, agricultural assets, household items, such as radio, television, refrigerator, modern stove, etc.) and housing conditions (e.g., habitation type, type of roof and walls, source of lighting, type of toilet and sanitation, water source in the dry and rainy season, energy source for cooking) [53]. The choice of quartiles rather than quintiles was largely arbitrary (both are common choices) but we chose quartiles to reduce small numbers in individual strata.

We used a binary variable indicating if there is any member in the household who has a non-agricultural occupation to adjust for any differences in the association of food energy from crop production and children’s MUAC related to income from other employment.

We used an indicator of whether a household had any garden produce, i.e., vegetables, fruits, and herbs, to adjust for food energy and nutrients households produced in addition to their cereal crop harvest or additional income that could have been generated from garden produce sales.

Data on village characteristics (presence of health care, education, and administrative facilities, markets, as well as the quality of roads and water wells) were obtained from the geographical information system database of the CSRN. From these we constructed a variable indicating the level of village infrastructure development, using principal components analysis of the variables just listed, and recoding the variable into quartiles. Verbal informed consent was obtained in all data collection from human subjects in agreement with the local community and with the approval of the Observational Ethics Committee and the Comité Institutionnel d’Ethique du Centre de Recherche en Santé de Nouna. Our study was also approved by the London School of Hygiene and Tropical Medicine Observational Ethics Committee.

### Analyses

The main analyses were made of MUAC as a continuous measure, but for descriptive statistics MUAC was also classified using the cut off values of 125 and 115 mm indicative of moderate and severe acute undernutrition [45].

The association of children’s nutritional status (MUAC) with the two measures of household crop harvest (E_f_ and E_fc_) was examined using multilevel regression models, accounting for clustering at the village level. Additionally, we examined interaction of these associations with children’s sex.

We used two methods of model-fitting: (i) restricted natural cubic splines (one internal knot placed at the median value of the E_f_ or E_fc_ [54]) to show variation in MUAC as a smooth function of E_f_ or E_fc_ and (ii) a piecewise linear regression model with a single change point below which MUAC was assumed to have a linear relationship with E_f_ or E_fc_, zero gradient was assumed above the change point. The latter models were fitted to be able to represent the relationship between MUAC and E_f_ or E_fc_ as a single regression slope. Akaike Information Criterion (AIC) and Likelihood Ratio (LR) tests were used to assess model fit including the number of knots for the restricted natural cubic spline models.

For the piecewise linear regression models we specified the change point a priori at 2900 kcal/ae/d, which corresponds to the recommended energy intake for a moderately active adult. For consistency, we used the same change points for piecewise models where E_f_ and E_fc_ were specified as the exposure.

All regression models were adjusted for potential confounders [55], namely: age, sex, month of MUAC measurement, the household wealth index, a village-level indicator of infrastructure development, and binary indicators of: participation of any household member in a non-agricultural occupation, whether the household had any garden produce, and a village-level indicator of the presence of a market. We also included an indicator of whether any of the crop types cultivated by the households in the year of study failed to provide any harvest to see if model results were sensitive to this adjustment. For transparency, we present the model results after adding selected groups of confounders until the full model with all confounders included.

Sensitivity analyses were undertaken to assess the impact of the exclusion of observations from households with high crop production values (>8000 kcal/ae/d from food crop harvest and >15,000 kcal/ae/d from food and cash crop harvest combined) – see Additional file 1: Table S2. Statistical analyses were carried out in Stata 14.1 [56].

## Results

Characteristics of the study population are given in Table 1. Nearly 50% of children in our analyses were <6 months of age because of the survey methods which targeted such children. The mean household size was 11 people. 31% of the households had at least one member involved in a non-agricultural occupation (such as pottery, brick making, trade, or other income-generating activity).

**Table 1:** Characteristics of households, children, and villages

Characteristics	Mean (25^th^, 50^th^, 75^th^ centile) or counts (%)
Household characteristics (*n* = 545)	
No. of people	11 (6, 9, 14)
Adult equivalents	8 (4, 7, 10)
Wealth	
Level 1 (poorest)	122 (23%)
Level 2	136 (25%)
Level 3	138 (26%)
Level 4 (wealthiest)	104 (19%)
Unclassified	45 (8%)
At least one member with occupation outside agriculture	167 (31%)
Garden produce harvested	383 (70%)
Cash crops harvested	431 (79%)
Food crops harvested	542 (99%)
Crop yield (kg/ae/year)^a^	
Millet	144 (53, 113, 190)
Sorghum	169 (70, 131, 208)
Maize	41 (6, 18, 42)
Fonio	14 (0, 5, 18)
Rice	18 (0, 0, 24)
Cotton	46 (0, 0, 56)
Sesame	61 (14, 33, 68)
Peanut	37 (5, 21, 53)
Food energy equivalent (kcal/ae/d):	
food crops	2978 (1609, 2493, 3769)
< 2900 kcal/ae/d	321 (59%)
food & cash crops	4213 (1965, 3211, 5483)
< 2900 kcal/ae/d	238 (44%)
Children’s characteristics (*n* = 975)	
Age	
0 − <6 months	464 (48%)
6 months − <2 years	222 (23%)
2 years − 5 years	289 (30%)
Sex	
Male	476 (49%)
Female	499 (51%)
MUAC^b^	135 (130, 130, 140)
< 115 mm	98 (10%)
115–125 mm	137 (14%)
Month of MUAC measurement	
March	131 (13%)
April	133 (14%)
May	208 (21%)
June	265 (27%)
July	139 (14%)
August	99 (10%)
Village characteristics (*n* = 52)	
Infrastructure level	
Level 1 (lowest)	32 (62%)
Level 2	14 (27%)
Level 3	4 (8%)
Level 4 (highest)	2 (4%)
Has a market	20 (37%)

*Abbreviations: MUAC* middle-upper arm circumference, *kcal/ae/d* kilocalories per adult equivalent per day

^a^0 production values present when crop was not cultivated or its harvest failed

^b^MUAC data in the table is presented for all children included in the analyses, aged 0**–**5 years. Corresponding number and (%) of children aged 6 months–5 years with MUAC < 115 mm was 16 (3%) and with MUAC 115–125 mm 51 (10%), which indicate the proportion of severely and moderately acutely malnourished children among our study subjects

Villages varied in the level of their infrastructural development assessed by the presence/absence of administrative, educational and medical facilities, market, transport, and water infrastructure. A market was present in only 37% of them.

**Fig. 2:**
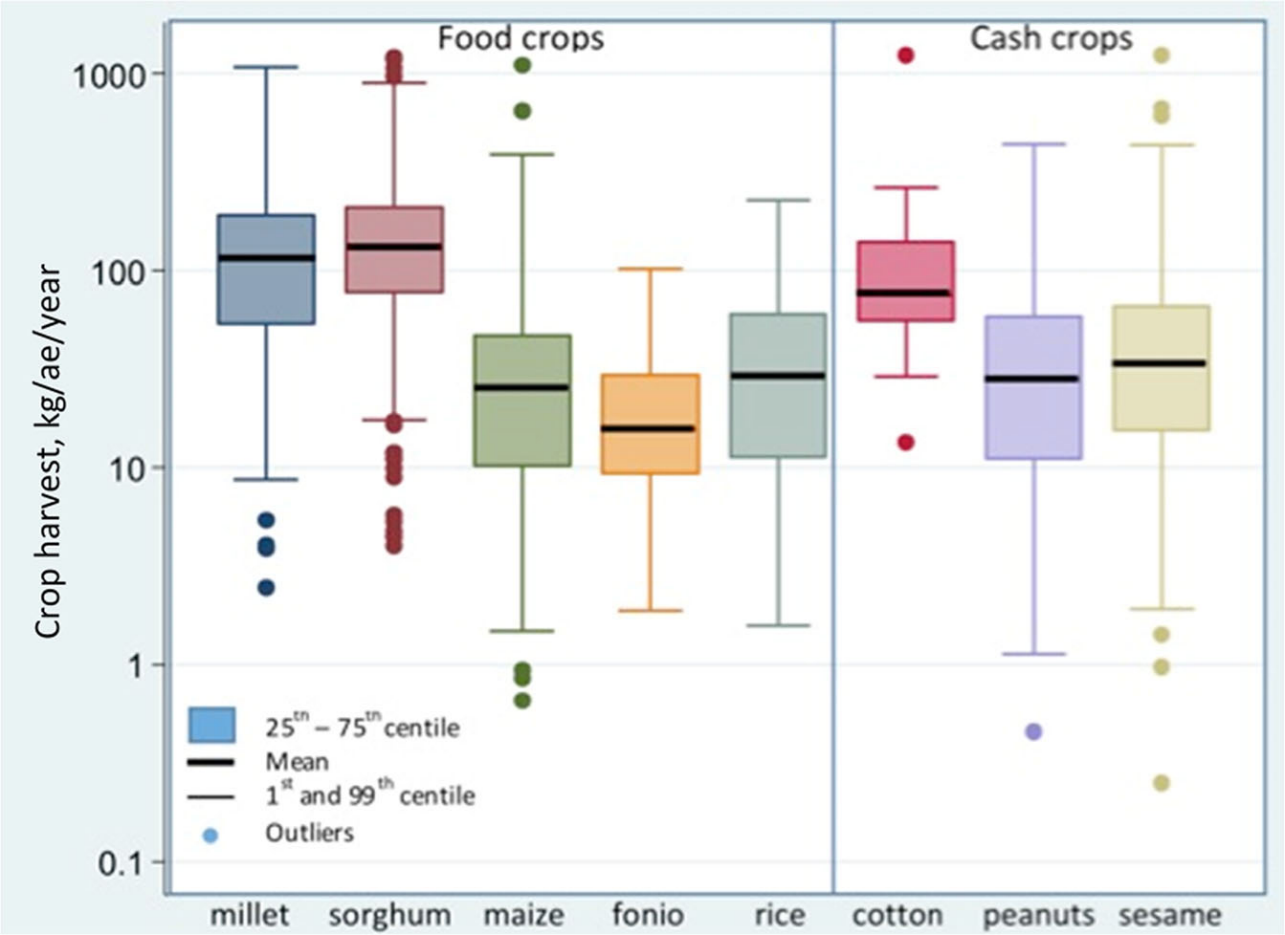
The amount and variability of crop harvest across households in the year 2009. Abbreviations: kg/ae/d, kilograms per adult equivalent per day. Note 1: Results are presented on a logarithmic (to the base of 10) scale. Note 2: Households with 0 production of the crop were excluded from data presented in this figure (but included in analysis) to demonstrate harvest variability among households that managed to produce the crop

Most of the crop produce in the year 2009 was derived from millet and sorghum (Table 1; Fig. 2). The harvest size varied considerably across households and crop types (Fig. 2). 70% of the households produced some garden produce, such as vegetables, fruit, and herbs (Table 1).

Although mean food energy value of household food crop harvest exceed the recommended kcal intake of 2900 kcal per day for moderate activity levels of our assumed adult equivalent (a 30–60 years old male) (Table 1), 62% of the children lived in households that produced less food energy from their food crop harvest than was recommended for their households for moderate activity levels and 55% less than recommended for light activity [51]. 32% of the children lived in households that produced less food energy from their combined food and cash crop harvest than was recommended for their households for moderate activity and 25% less than recommended for light activity [51].

Average MUAC among children of 0–5 years of age was 135 mm with 10% of the children having MUAC <115 mm and 14% between 115 & 125 mm. Among children aged 6 months–5 years, 3% had MUAC <115 mm and 10% MUAC between 115 & 125 mm, indicating the proportions of severe and moderate acute undernutrition respectively (Table 1).

### Relationship between MUAC and crop harvests

**Fig. 3:**
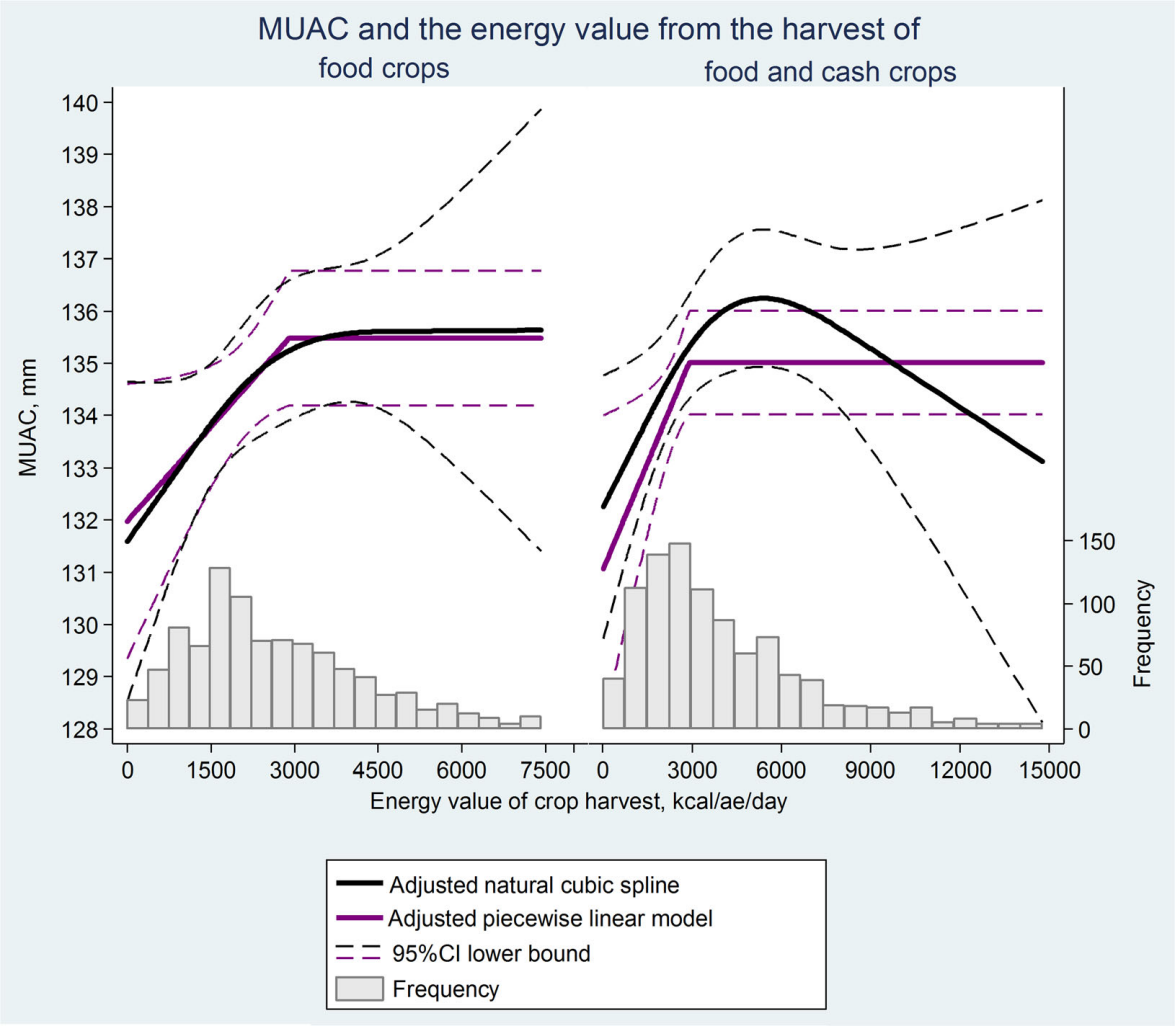
Restricted natural cubic spline and piecewise linear models of the associations of children’s MUAC with food energy production. On the left: food energy estimates are based on food crop harvest alone. On the right: food energy estimates are based on food and cash crop harvest combined. Abbreviations: CI, confidence interval; kcal/ae/d, kilocalories per adult equivalent per day; MUAC, middle-upper arm circumference; HDSS, Health and Demographic Surveillance System. Note 1: For purposes of clarity in the visual presentation, we excluded from the models the sparse observations at the highest exposure values: 40 observations excluded within 8000–16,052 kcal/ae/day interval of the energy production from food crop harvest and 14 observations were excluded within 15,000–34,064 kcal/ae/day interval of the energy production from both food and cash crops combined. Note 2: Both models were adjusted for age, sex, month of MUAC measurement, household wealth, non-agricultural occupation, garden produce, village infrastructure, and market presence

The relationships between MUAC and the two measures of annual household per capita crop harvest (kcal/ae/d), E_f_ and E_fc_, are shown in Fig. 3. These plots suggest that children’s MUAC decreased at crop yields below around 3000 kcal/ae/d. The children’s MUAC was 2.49 (95% CI 0.45, 4.52) mm less at 1000 than at 3000 kcal/ae/d when food energy estimates were based on cereal food crop production alone (E_f_), and 1.99 (95% CI 0.27, 3.69) mm less when food energy estimates were based on food and cash crop production combined (E_fc_) (Table 2).

**Table 2:** Estimated differences in MUAC (mm) per difference in food energy production from crop harvest

Model adjustments	Reduction in MUAC (95% CI) at 3000 *vs* 1000 kcal/ae/d^a^:		Reduction in MUAC (95% CI) for a 1000 kcal/ae/d decline in crop harvest below 2900 kcal/ae/d^b^:	
	Food crop harvest alone	Food & cash crop harvest combined	Food crop harvest alone	Food & cash crop harvest combined
Model 1: unadjusted	2.44 (0.14, 4.73)	2.01 (0.12, 3.90)	1.27 (−0.01, 2.55)	1.64 (0.22, 3.05)
Model 2: adjusted for children’s age, sex, and month of MUAC measurement	2.67 (0.58, 4.76)	1.98 (0.26, 3.71)	1.44 (−0.27, 2.60)	1.57 (0.29, 2.85)
Model 3: model 2 + adjustments for household wealth, non-agricultural occupation, garden produce, village infrastructure, and market presence	2.49 (0.45, 4.52)	1.99 (0.27, 3.69)	1.29 (0.15, 2.42)	1.55 (0.30, 2.81)
Model 4: model 3 + adjustment for failure to harvest at least one of the cultivated crops	2.49 (0.46, 4.53)	1.97 (0.26, 3.67)	1.29 (0.15, 2.43)	1.57 (0.31, 2.83)

*Abbreviations: CI* confidence interval, *MUAC* middle-upper arm circumference, *kcal/ae/d*, kilocalories per adult equivalent per day

^a^Estimates based on models with natural cubic splines

^b^Estimates based on piecewise linear models

Piecewise linear models with a change point at 2900 kcal/ae/d suggest that below 2900 kcal/ae/d, MUAC decreased by 1.29 (CI 95% 0.15, 2.42) mm per 1000 kcal/ae/d decrease in household food energy production from cereal food crops only (E_f_), and by 1.55 (CI 95% 0.30, 2.81) mm per 1000 kcal/ae/d decrease in food energy from food and cash crop harvest combined (E_fc_) (Table 2).

These results were largely insensitive to the exclusion of observations with high crop production values (see Additional file 1: Table S2) and the additional adjustment for crop failure (last line of Table 2).

There was no clear evidence that the decrease in MUAC with lower household food crop yields was different in boys *vs* girls (Table 3, *p* = 0.203 for statistical interaction), but the point estimates of the decrease were larger in boys. Boys’ MUAC was 3.81 (95% CI 0.84, 6.77) mm lower at 1000 than at 3000 kcal/ae/d when food energy estimates were based on cereal food crop production alone (E_f_), and 3.47 (95% CI 0.97, 5.96) mm less when food energy estimates were based on food and cash crop production combined (E_fc_). The corresponding figures for girls were 0.99 (95% CI −1.81, 3.78) mm, and 0.86 (95% CI −2.13, 2.57) mm.

**Table 3:** Adjusted estimates of differences in MUAC (mm) per specified difference in food energy production from crop harvest by sex

Sex	No. (%)	Mean MUAC, mm	Mean food energy from food crop harvest, kcal/ae/d	Difference in MUAC (95% CI): 3000 *vs* 1000 kcal/ae/d^a^		Reduction in MUAC (95% CI) for a 1000 kcal/ae/d decline in crop harvest below 2900 kcal/ae/d^b^:		
				Food crop harvest alone	Food & cash crop harvest combined	Food crop harvest alone	Food & cash crop harvest combined	*p*-value for interaction^c^
All children	975 (100)	135	2978	2.49 (0.45, 4.52)	1.99 (0.27, 3.69)	1.29 (0.15, 2.42)	1.55 (0.30, 2.81)	
Boys	499 (51)	136	3094	3.81 (0.84, 6.77)	3.47 (0.97, 5.96)	2.15 (0.49, 3.80)	2.43 (0.56, 4.30)	0.203
Girls	476 (49)	133	2842	0.99 (−1.81, 3.78)	0.86 (−2.13, 2.57)	0.42 (−1.14, 1.99)	0.69 (−1.02, 2.40)	

*Abbreviations*: *CI* confidence interval, *MUAC* middle-upper arm circumference, *kcal/ae/d* kilocalories per adult equivalent per day, *LRT* likelihood ratio test

^a^Estimates based on models with natural cubic splines

^b^Estimates based on piecewise linear models

^c^ *p*-value presented for the LRT of interaction applied to the piecewise model with the exposure of food energy from food crop production

In line with these results, Additional file 1: Table S3 shows that the prevalence of acute undernutrition (MUAC <125 mm) was slightly higher in households with ≤2900 kcal/ae/d food cereal crop energy production. Such households also had lower diversity of crops (as reflected in the number of different crops harvested) and they less frequently produced cash crops and garden produce. Their household size was slightly larger, and they more frequently had at least one member of their household involved in a non-agricultural occupation.

## Discussion

This is one of the few studies examining the association of children’s nutritional status with household cereal crop production. Its results suggest that low household production of food energy from cereal crops is associated with lower MUAC for children ≤5 years of age.

The results of the restricted spline plot suggest a decline in MUAC below around 3000 kcal/ae/d, which is broadly consistent with the recommended energy intake of 2900 kcal/d for a moderately active man of 30–60 years of age [51, 52]. The results of the linear spline model show a statistically significant decline in MUAC very similar in gradient to that of the restricted spline plot when the change point was fixed a priori at 2900 kcal/ae/d.

Our findings are consistent with some [22–24] but not all [20, 21] studies examining the association between children’s nutritional status and household level measures of agricultural production. Variation in findings across different studies may be explained by different contexts [20], choice of nutritional status measures and temporality of their association with crop harvest (e.g., acute *vs* chronic undernutrition [20]), and other modifying factors.

We did not find clear evidence of a gender difference in the association of crop production with children’s nutritional status. Our findings suggest possibly more pronounced association among boys than girls. This could in part be related to the higher level of child undernutrition among boys than girls in our setting [57]. Given the relatively small study sample, we cannot conclude a difference, but the point estimates were larger in boys than girls.

The current study was conducted in a population whose livelihood is likely to be particularly vulnerable to crop failure and low cereal crop productivity. Over a half of the examined children lived in households whose food crop production in the year 2009 was not sufficient to meet their energy needs for even light activity. A quarter of households would not be able to reach their energy requirements for light activity levels even when selling all their cash crops and purchasing millet instead. The association between low levels of household crop harvest and acute child undernutrition is highly plausible in such context.

We must note that the MUAC measurements analysed in this study were made in the six nutritionally more challenging months of the year in the study area, as they include the period when household cereal stocks from the last harvest start to run low (the time often referred to as the ‘lean’ or the ‘hunger’ season) [58]. Analysis of MUAC data collected evenly throughout the year may yield a lower magnitude of the examined association.

The high proportion (14%) of acute undernutrition among children in our study population is of serious public health significance, according to the guidelines of the World Health Organization (WHO) [59]. The prevalence of acute undernutrition above 10% is not uncommon in many low- and middle-income countries in Africa, South and South-East Asia [60]. Similar analyses of the association between household crop production and children’s nutritional status in other countries of these regions would help identifying whether household crop production levels in these settings also incur risk for their children’s nutritional health.

Our study population and similar populations may be vulnerable to the adverse effects of climate change on agricultural productivity. Given that the association between children’s nutritional status and household crop harvest was identified even in a year of average agricultural productivity and given the evidence of the link between weather-related area-wide crop failures with negative nutritional outcomes among children in similar settings [30, 31, 61–66], it is likely that the Nouna HDSS population would experience greater levels of acute child undernutrition in years of low agricultural productivity. Droughts are already recognised as the top natural disaster in Burkina Faso and their frequency and severity is projected to increase with climate change, potentially leading to increased episodes of low crop yields [67–69].

However, our estimates were based on harvest differences across households in a single year with average crop productivity. According to Annual National Agricultural Survey data, over the five years preceding our examined harvest year in the Kossi province did not fall below the average yield level calculated over the period of 1984–2014 [70]. Therefore, our estimated magnitude of change in children’s MUAC per difference in food energy from household crop harvest is applicable to average yield levels, and hence, should not be used to infer the possible change in children’s MUAC in response to inter-annual changes in household harvest, particularly those resulting from drought or other exogenous shocks. However, the identified association in a year of average crop productivity does suggest the likely vulnerability of our study population to weather-related and other declines in crop yield.

We used the indirect measure of food energy production from crop harvest to approximate household food energy availability. Our measure did not take into account other food sources possibly acquired by households (e.g., food purchases, gifts, and loans [71]) or disposal of the produce (e.g., transfers to others and food waste). Apart from household food energy availability, children’s food intake is subject to intra-household food distribution and children’s food preferences; children’s nutritional status, apart from food intake, is also determined by their health condition and other factors [4]. Furthermore, we used household harvest data reported by the household head. In the socio-cultural context of our study, the household head is the key informant on the amount of the household’s crop harvest. However, reported data (as opposed to quality controlled measurements or observations made by data collectors, which in our study area were not available) may have some inconsistencies.

In our analyses, it was not possible to account for crop harvest households produced in years preceding or following the year 2009, as there were no data collected. Such information could help adjusting for any effect of crop harvest on children’s MUAC prior to the year 2009 and for any cereal stock remaining from a preceding year that households could consume in addition to the crops harvested in the year 2009. However, differences in child MUAC in the year 2010 related to household crop harvest in years preceding 2009, if any present at all, would be minor, as MUAC is sensitive to short-term changes in food intake. The influence of previous harvests on our examined MUAC measurements was likely to have been superseded by the influence of the harvest of the year 2009. The harvest of 2010 could have some influence on food intake in August 2010, if any of the households started the harvest of 2010 earlier than September, as assumed in our analyses. This was also impossible to account for due to the lack of information on when individual households started their harvest in the year 2010.

Other minor limitations include: (1) our adult equivalent calculations were based on the number of household members at the time of the harvest of 2009, and hence, did not account for any possible changes in household composition between the harvest time and the time of child MUAC measurement (March–August 2010), (2) food price estimates were based on a single time point (December 2009, when crop sales occur frequently) for the Nouna market, as the largest market in the study area [72], hence, we did not account for any fluctuations in food price across the year.

## Conclusion

MUAC measurements made during the months of March–August following a ‘normal’ harvest year, indicate negative impacts of low household cereal crop yields on child nutrition in this rural subsistence farming population of Burkina Faso.

The results suggest that this and similar populations may be adversely affected by low levels of crop harvest and vulnerable to the adverse effects of weather and other factors on household crop yields, especially in the context of climate change. Nutrition-sensitive monitoring of household crop yields and support provision to the most vulnerable households in such settings could aid the achievement of the SDG No. 2 in the face of the projected climate change impacts on agricultural productivity.

## Additional file

Additional file 1: (1) Note on study subject representativeness, (2) Sensitivity Analysis I: exclusion of observations with high crop production values, (3) Characteristics of study population in relation to food energy production from food crops. (DOCX 44.8 kb)

## Abbreviations

AIC: Akaike information criterion
CRSN: Centre de Recherche en Santé de Nouna
E_f_: Energy value of average daily household food cereal crop produce
E_fc_: Energy value of average daily household food and cash crop produce combined
HDSS: Health and demographic surveillance system
IQR: Interquartile range
kcal/ae/d: Kilocalories per adult equivalent per day
LR: Likelihood ratio
MUAC: Middle-upper arm circumference
SDG: Sustainable Development Goal
SOFITEX: Societé Burkinabé des Fibres et Textiles
WHO: World Health Organization
