# Supplementary material for: Household cereal crop harvest and children’s nutritional status in rural Burkina Faso
Source: Environ Health. 2017 Jun 20;16:65. doi: 10.1186/s12940-017-0258-9 (PMC5477741; doi:10.1186/s12940-017-0258-9)
Supplement: Additional file 1: — (1) Note on study subject representativeness, (2) Sensitivity Analysis I: exclusion of observations with high crop production values, (3) Characteristics of study population in relation to food energy production from food crops. (DOCX 44.8 kb) [file 12940_2017_258_MOESM1_ESM.docx]

Additional file 1

HOUSEHOLD CEREAL CROP HARVEST AND CHILDREN’S NUTRITIONAL STATUS IN RURAL BURKINA FASO

Belesova, K., Gasparrini, A., Sié, A., Sauerborn, R., Wilkinson, P.

Table of contents

Note on study subject representativeness.………………………………………….……………....…S2

Sensitivity Analysis I: exclusion of observations with high crop production values.....………..…….S3

Characteristics of the study population in relation to food energy production from food crops…...…S4

**Note on study subject representativeness**

Although our study subjects were not randomly selected from the Nouna HDSS population, their demographic and socio-economic characteristics, except age, did not differ from the characteristics of children 0–5 years of age in all Nouna HDSS population, as recorded during the census of the year 2009 (Table S1).

**Table S1. Characteristics of the study subjects *vs* population of children ≤5 years of age in the Nouna HDSS system.**

|  | Study subjects  % (n) | Population (census)  % (n) |
| --- | --- | --- |
| Children’s characteristics | **n=975** | **n=17,112** |
| Age  0 – <6 months  6 months – <2 years  2 years – 5 years | 48 (464)  23 (222)  30 (289) | 6 (1,009)  29 (4,916)  65 (11,187) |
| Sex  Females  Males | 51 (499)  49 (476) | 50 (8,518)  50 (8,594) |
| Household characteristics | **n=545** | **n=7,695** |
| At least one member with occupation outside agriculture | 31 (301) | 34 (2,638) |
| Wealth  Level 1 (poorest)  Level 2  Level 3  Level 4 (wealthiest)  Unclassified | 23 (122)  25 (136)  26 (138)  19 (104)  8 (45) | 22 (1,727)  22 (1,687)  22 (1,690)  20 (1,544)  14 (1,047) |
| Village-level characteristics | **n=52** | **n=59** |
| Village infrastructure  Level 1 (lowest)  Level 2  Level 3  Level 4 (highest) | 62 (32)  14 (14)  8 (4)  4 (2) | 63 (37)  27 (16)  8 (4)  3 (2) |
| Has a market | 37 (20) | 37 (22) |

### Abbreviations: HDSS, Health and Demographic Surveillance System; n, number.

**Sensitivity Analysis I: exclusion of observations with high crop production values**

| **Model adjustments** | **Reduction in MUAC (95% CI)**  **at 3,000 *vs* 1,000 kcal/ae/d ^a^:** | | **Reduction in MUAC (95% CI) for a 1,000 kcal/ae/d decline in crop harvest below 2,900 kcal/ae/d ^b^:** | |
| --- | --- | --- | --- | --- |
|  | **Food crop harvest alone** | **Food & cash crop harvest combined** | **Food crop harvest alone** | **Food & cash crop harvest combined** |
| Model 1: unadjusted | 2.63 (–0.07, 5.94) | 2.19 (0.11, 4.28) | 1.26 (–0.03, 2.56) | 1.69 (0.27, 3.11) |
| Model 2: adjusted for children’s age and sex, and month of their MUAC measurement | 2.70 (0.24, 5.16) | 2.22 (0.33, 4.11) | 1.42 (0.24, 2.61) | 1.60 (0.31, 2.88) |
| **Model 3: model 2 + adjustments for household wealth, non-agricultural occupation, garden produce, village infrastructure, and market access** | **2.49 (0.09, 4.89)** | **2.34 (0.47, 4.22)** | **1.29 (0.13, 2.45)** | **1.56 (0.29, 2.82)** |
| Model 4: model 3 + adjustment for failure to harvest at least one of the cultivated crops | 2.50 (0.11, 4.91) | 2.35 (0.48, 4.22) | 1.29 (0.13, 2.45) | 1.57 (0.30, 2.84) |

This sensitivity analysis assessed the impact of the exclusion of observations with high crop production values (>8,000 kcal/ae/d from food crop harvest and >15,000 kcal/ae/d from food and cash crop harvest combined), which are implausible for the scale of subsistence farmers’ production. These exclusions were also made for the purpose of clarity in the visual presentation of the Figure 2.

**Table S2. Estimated differences in MUAC (mm) (95% CI) per difference in food energy production from crop harvest.**

Abbreviations: AIC, Akaike Information Criterion; CI, confidence interval; MUAC, middle-upper arm circumference; kcal/ae/d, kilocalories per adult equivalent per day.

^a^ Estimates based on the natural cubic splines.

^b^ Estimates based on piecewise linear models; presented are model estimates for the interval of food energy <2,900 kcal/ae/d.

**Table S3. Characteristics of the study population in relation to food energy production from food crops.**

| Characteristics | Counts (column %) or median (IQR) | |
| --- | --- | --- |
|  | **≤**2,900 kcal/ae/d | >2,900 kcal/ae/d |
| Household production related characteristics ^a^ | | |
| Cash crops harvested | 231 (72) | 200 (89) |
| Garden produce harvested | 207 (64) | 176 (79) |
| No. of different crops harvested  1  2  3  4  5  6  7 | 37 (12)  71 (22)  86 (27)  73 (23)  44 (14)  8 (3)  2 (1) | 9 (4)  17 (8)  57 (26)  77 (34)  33 (15)  27 (12)  4 (2) |
| Other household characteristics ^a^ | | |
| No. of people | 11 (7, 16) | 8 (5, 12) |
| Adult equivalents | 8 (5, 12) | 6 (3, 8) |
| At least one member with occupation outside agriculture | 115 (35) | 52 (23) |
| Wealth  Level 1 (poorest)  Level 2  Level 3  Level 4 (wealthiest)  Unclassified | 72 (22)  83 (26)  76 (24)  71 (22)  20 (6) | 52 (23)  53 (24)  64 (29)  36 (16)  18 (8) |
| Village-level characteristics ^a^ | | |
| Village infrastructure  Level 1 (lowest)  Level 2  Level 3  Level 4 (highest) | 117 (36)  72 (22)  40 (12)  92 (29) | 77 (34)  68 (30)  34 (15)  45 (20) |
| Have a market in their village | 205 (64) | 143 (64) |
| Village  Nouna  Kodougou  Bagala  Dara  Ley | 78 (24)  17 (5)  4 (1)  3 (1)  2 (1) | 29 (13)  2 (1)  2 (1)  4 (2)  1 (1) |
| Children's characteristics ^b^ | | |
| Age  0 – <6 months  6 months – <2 years  2 years – 5 years | 272 (48)  130 (23)  169 (30) | 192 (48)  92 (23)  120 (30) |
| Sex  Females  Males | 273 (48)  298 (52) | 226 (56)  178 (44) |
| Nutritional status ^c^  MUAC<115mm  115mm≤MUAC<125mm | 7 (2)  36 (13) | 9 (4)  15 (7) |

###

### Abbreviations: kcal/ae/d, kilocalories per adult equivalent per day; IQR, inter quartile range; MUAC, middle-upper arm circumference.

### ^a^ Statistics in this section of the table are based on household-level observations (n=545)

### ^b^ Statistics in this section of the table are based on child-level observations (n=975)

### ^c^ Counts (%) based on observations of children of 6 months–5 years of age, the age group where the presented MUAC cut-off values are used to determine the states of severe (MUAC<115 mm) and moderate (MUAC 115–125 mm) acute malnutrition.
